# Botulinum Toxin Injection for Painful Adductor Pollicis Contracture after Thumb Carpometacarpal Resection Arthroplasty

**DOI:** 10.3390/life12010110

**Published:** 2022-01-13

**Authors:** Matthias Holzbauer, Gerhard Großbötzl, Stefan Mathias Froschauer

**Affiliations:** 1 Department of Traumasurgery, Kepler University Hospital, Krankenhausstrasse 9, 4020 Linz, Austria; Matthias.holzbauer@a1.net; 2 Medical Faculty, Johannes Kepler University Linz, Altenbergerstr. 69, 4040 Linz, Austria; 3 Department of Orthopedics and Traumatology, Kepler University Hospital, Krankenhausstrasse 9, 4020 Linz, Austria; gerhard.grossboetzl@kepleruniklinikum.at

**Keywords:** adductor pollicis contracture, botulinum toxin, carpometacarpal joint, spasmodic pain

## Abstract

Pollux adductus deformity is an accompanying symptom of thumb carpometacarpal osteoarthritis. We describe a case of a patient who presented with increased muscle tone of the adductor pollicis muscle and chronic pain in the thenar musculature, i.e., recurrence of an adduction deformity. The patient reported a symptom-free period of 5.5 years after having received resection-suspension-arthroplasty for stage IV thumb carpometacarpal osteoarthritis until spasmodic pain appeared. Due to the functional impairment of this condition, we administered therapy including 100 units of Botox^®^ (onabotulinumtoxinA, Allergan, Dublin, Ireland) injected with a fanning technique into the adductor pollicis muscle. Thus, we observed a substantial improvement in the patient-reported outcome measures as well as pain levels compared with initial values. The current case shows the pivotal role of the adductor pollicis muscle when patients report pain at the base of the thumb, which can cause considerable impairments despite the complication-free surgical treatment of thumb CMC OA.

## 1. Introduction

Adduction, or an “M” deformity, is a well-known symptom of thumb carpometacarpal (CMC) osteoarthritis (OA) [1]. Pathophysiologically, this sign is caused by hypertonia of the adductor pollicis (AP) muscle, which is a suspected protective mechanism to stabilize the damaged CMC joint and reduce arthritis-related complaints [2]. Over the course of time, this leads to contracture of the AP muscle or the whole fist web; hence, the range of motion (ROM) of the thumb CMC joint is impaired. This sign is associated with an advanced stage of thumb CMC OA [1]. Generally, this condition is associated with a typical history: patients commonly report pain localized at the base of the thumb radiating to the thenar eminence [3,4]. This pain is aggravated during activity, especially involving forceful pinching. The grind test—in addition to some modified clinical tests, e.g., the distraction test, pressure-shear test, metacarpal base compression test, thumb adduction stress test, extension stress test, and thumb flection stress test—is the most commonly applied clinical test to reproduce this pain to confirm the diagnosis of thumb CMC OA [5]. In a further step, radiographs primarily aid staging of the disease [1]. 

The current case report concerns a female patient who presented with an AP contracture with typical pain after having received a resection-suspension-arthroplasty 6 years prior. The patient showed a symptom free interval of 5.5 years with physiological ROM and no OA of an adjacent joint; therefore, we proceeded with a therapeutic approach using botulinum neurotoxin injection into the AP muscle. Moreover, due to the absence of an arthritically degenerated thumb CMC joint, the systematical follow-up of this patient represented an examination of the isolated impact of the AP muscle on symptoms associated with an adduction deformity. 

## 2. Case Presentation

We present a case of a 59-year-old female patient with spasmodic pain radiating along the palmar eminence, with its strongest intensity located between the proximal part of the first and second metacarpal after surgical treatment for thumb CMC OA. The patient described these symptoms as dragging pain or as spasm in the thenar musculature, which was aggravated by manual work and could be reduced or even relieved if she intensely massaged this region. She had compared the localization and modality of this pain with that experienced preoperatively. The patient had received resection-suspension-arthroplasty using the modified Martini technique for symptomatic, Eaton–Littler stage IV thumb CMC OA 6 years prior [6]. This procedure included extensive soft tissue release at the base of the first metacarpal because of a progredient AP contracture. Six weeks after this procedure, the patient started performing intensive hand therapy for 6 months; hence, she regained physiological ROM and was symptom-free for an overall of 5.5 years.

Physical examination at presentation revealed no swelling, signs of carpal tunnel syndrome, and a negative grind test. However, we could detect a moderate recurrence of AP contracture with increased muscle tone combined with hyperextension of the thumb metacarpophalangeal joint to 15°. ROM assessment, especially radial abduction, as well as grip and pinch strength, showed decreased values (see Table 1) [7]. Overall, this clinical presentation resembled the status before initial surgery. 

Moreover, patient-reported outcome measures (PROMs), as well as pain levels rated using a visual analogue scale (ranging from 0 to 10), showed a relatively high, subjective impairment of this condition, i.e., spasmodic pain (see Table 1). 

Radiographic assessment showed no signs of thumb shortening or OA of adjacent wrist joints. 

**Figure 1:**
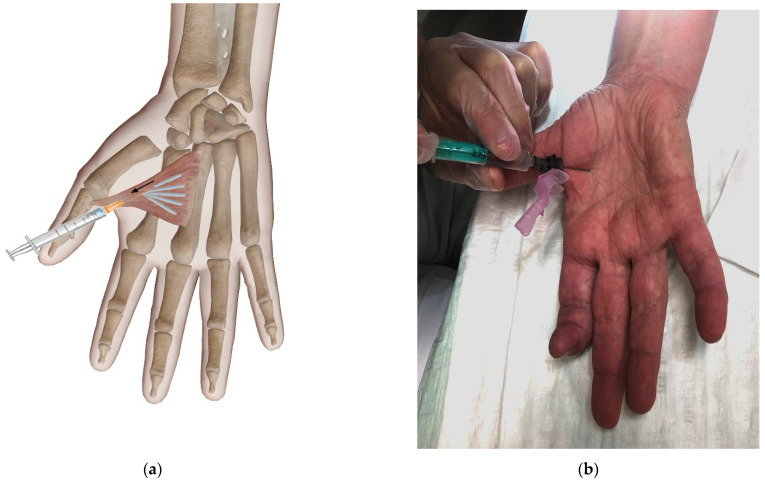
Botox injection using a fanning technique displayed as a schematic drawing (**a**) image courtesy of Visible Body and (**b**) a photograph of the patient involved in the present case report.

Due to the subjective complaints associated with high functional impairment and the clinical presentation, we suspected AP muscle contracture to be the morphologic cause of this condition. Thus, we decided to perform a therapy including an injection of Botox^®^ (onabotulinumtoxinA, Allergan, Dublin, Ireland) into this muscle. In detail, we used 100 units injected with a fanning technique at a flat angle (see Figure 1).

To monitor the effect of this novel, therapeutic approach, we conducted follow-up assessments 2 weeks, 3 months, and 6 months after this injection (see Table 1). According to the PROMS, pain levels and functional outcome values, it was observed that considerable improvements in this condition could be achieved up to a minimum period of 3 months postinterventionally. Six months after the injection, the outcome parameters approximately reached the initial values.

## 3. Discussion

The clinical and PROM outcomes show that substantial improvements in the symptoms could be achieved after 3 months by injecting botulinum neurotoxin into the AP muscle. Especially, pain levels could be considerably reduced, which led to subjective satisfaction of the patient. The duration of action is in accordance with previous studies reporting an average duration of continuous Botox^®^ effects being between 3 and 4 months [8,9]. 

In addition to muscle relaxation caused by the agent’s effects at the local synaptic terminal, there is an ongoing discussion in the medical literature that pain relief is mediated via other mechanisms [9]. For example, intramuscularly administrated botulinum neurotoxin can lead to prolonged pain reduction in comparison to the duration of muscle-relaxant effects [10]. A potential explanation might be a distinct secondary uptake pathway, involving retrograde transport to the dorsal root ganglion and the spinal cord, which is considered to play a pivotal role in pain relief [11]. 

Although botulinum neurotoxin was originally used in neuromuscular disorders for its paralyzing effects, it is increasingly used in musculoskeletal conditions, e.g., plantar fasciopathy, OA, lateral epicondylitis and myofascial pain syndrome [12]. Regarding the thumb CMC joint, the medical literature currently only contains a single report concerning botulinum toxin: one preliminary published study protocol involved intra-articular injection [13]. To the best of our knowledge, there are no reports available in treating painful AP muscle contracture. In addition to the successful symptom relief using botulinum neurotoxin injection in our particular case, the present report aims to emphasize the importance of the thenar musculature concerning thumb CMC OA. It is already known that exercise programs addressing muscular stabilization and proprioception of the joint yield better outcomes in conservative treatment of the primary condition [2]. However, these studies cannot prove whether functional improvements are caused by pain reduction related to the arthritically degenerated joint itself or related to the musculature. In other words, it is difficult to locate the morphologic origin of the main pain. The current case provided the opportunity to study the effect of increased muscle tone of the AP muscle in the absence of any arthrosis pain. Thus, we could detect a considerable impact of AP muscle contracture on the symptom complex related to adduction deformity: after paralyzing the AP using botulinum neurotoxin, we monitored pain via subjective (PROMs, pain level) and objective parameters (ROM and strength improvement), finding that this structure had a substantial impact on the patient’s complaints. Moreover, the recurrence of symptoms after 6 months, representing the period of time after which the action of Botox^®^ usually diminishes, additionally proves the causal link between the AP muscle and the symptoms.

Therefore, we hypothesize that too subordinate a role was attributed to the thenar musculature in thumb CMC OA, both during the primary condition as well as in the postoperative situation. Especially, AP contracture caused by an increased muscle tone might not simply be an accompanying symptom of thumb CMC OA, but rather a morphologic correlate of pain and functional impairment. 

A limitation of the present report is that our findings are based on a single patient and no control patient or group is available. Thus, we are planning a further study on the therapeutic effect of botulinum neurotoxin on painful AP contracture and its utility in the therapy regimen of thumb CMC OA. To prevent any potential harm of the recurrent motor branch of the median nerve [14], ultrasound-guided botulinum neurotoxin injection into the AP muscle will be performed in this future series of patients [15].

The current case shows that despite a complication-free, successful surgical treatment of thumb CMC OA, a considerable impairment can be caused by AP contracture. Despite the effects of this botulinum neurotoxin injection therapy being short-term, it can be valuable to achieve pain relief. Further studies should focus on the isolated effect of the AP on the symptom complex related to thumb CMC OA, which might yield new treatment approaches. 

## Figures and Tables

**Table 1:** Functional parameters including Disabilities of the Arm, Shoulder and Hand (DASH) score, Michigan Hand Outcomes Questionnaire (MHQ), and Patient-Rated Wrist Evaluation (PRWE) at baseline level and during follow-up examination after Botox^®^ injection.

Parameters	Preint.	2 W	3 M	6 M
DASH	56	34	39	59
MHQ	40	74	63	33
PRWE	67	29	39	57
Pain at rest	8	2	3	5
Pain at activity	8	2	2	5
Radial Abduction	30°	35°	35°	30°
Palmar Abduction	40°	45°	45°	40°
Opposition	0 cm	0 cm	0 cm	0 cm
Hand grip	15 kg	19 kg	21 kg	14 kg
Pinch grip	3.5 kg	4 kg	3.5 kg	3 kg

Preint., preinterventionally; W, weeks; M, months.

## Data Availability

All data are included in the manuscript.
